# Burosumab for X-linked hypophosphatemia in children and adolescents: Opinion based on early experience in seven European countries

**DOI:** 10.3389/fendo.2022.1034580

**Published:** 2023-01-31

**Authors:** M. Zulf Mughal, Giampiero I. Baroncelli, Carmen de Lucas-Collantes, Agnès Linglart, Andrea Magnolato, Adalbert Raimann, Fernando Santos, Dirk Schnabel, Nick Shaw, Ola Nilsson

**Affiliations:** ^1^ Department of Paediatric Endocrinology and Metabolic Bone Diseases, Royal Manchester Children’s Hospital, Manchester, United Kingdom; ^2^ The Faculty of Biology, Medicine, and Health, University of Manchester, Manchester, United Kingdom; ^3^ Division of Pediatrics, Endocrine Unit, ERN-BOND Representative, Department of Obstetrics, Gynecology and Pediatrics, University-Hospital, Pisa, Italy; ^4^ Servicio Nefrología, Hospital Infantil Universitario Niño Jesús, Universidad Autónoma de Madrid, Madrid, Spain; ^5^ AP-HP, Endocrinology and Diabetes for Children, Reference Center for Rare Disorders of Calcium and Phosphate Metabolism, Filière OSCAR, Bicêtre Paris Saclay Hospital, Paris, France; ^6^ Platform of Expertise for Rare Disorders, INSERM, Physiologie et Physiopathologie Endocriniennes, Paris Saclay University, Paris, France; ^7^ Department of Pediatrics, Institute for Maternal and Child Health – IRCCS “Burlo Garofolo”, Trieste, Italy; ^8^ Department of Pediatrics and Adolescent Medicine, Division of Pediatric Pulmonology, Allergology and Endocrinology, Medical University of Vienna, Vienna, Austria; ^9^ Vienna Bone and Growth Center, Vienna, Austria; ^10^ Hospital Universitario Central de Asturias (HUCA), University of Oviedo, Oviedo, Spain; ^11^ Center for Chronic Sick Children, Pediatric Endocrinology, Charitè, University Medicine, Berlin, Germany; ^12^ Department of Endocrinology and Diabetes, Birmingham Women’s and Children’s NHS Foundation Trust, Institute of Metabolism and Systems Research, University of Birmingham, Birmingham, United Kingdom; ^13^ Division of Pediatric Endocrinology, Department of Women’s and Children’s Health , Karolinska Institutet and University Hospital, Stockholm, Sweden; ^14^ School of Medical Sciences, Department of Pediatrics, Örebro University and University Hospital, Örebro, Sweden

**Keywords:** XLH, rickets, burosumab, children, adolescents, growth plate closure, dosing, transition

## Abstract

Given the relatively recent introduction of burosumab in the management of X-linked hypophosphatemia (XLH), there is limited real-world data to guide its use in clinical practice. As a group of European physicians experienced with burosumab treatment in clinical practice, we convened with the objective of sharing these practice-based insights on the use of burosumab in children and adolescents with XLH. We attended two virtual meetings, then discussed key questions *via* Within3, a virtual online platform. Points of discussion related to patient selection criteria, burosumab starting dose, dose titration and treatment monitoring. Our discussions revealed that criteria for selecting children with XLH varied across Europe from all children above 1 year to only children with overt rickets despite conventional treatment being eligible. We initiated burosumab dosing according to guidance in the Summary of Product Characteristics, an international consensus statement from 2019 and local country guidelines. Dose titration was primarily guided by serum phosphate levels, with some centers also using the ratio of tubular maximum reabsorption of phosphate to glomerular filtration rate (TmP/GFR). We monitored response to burosumab treatment clinically (growth, deformities, bone pain and physical functioning), radiologically (rickets and deformities) and biochemically (serum phosphate, alkaline phosphatase, 1,25-dihydroxyvitamin D, 25-hydroxyvitamin D, urine calcium-creatinine ratio and TmP/GFR). Key suggestions made by our group were initiation of burosumab treatment in children as early as possible, from the age of 1 year, particularly in those with profound rickets, and a need for clinical studies on continuation of burosumab throughout adolescence and into adulthood.

## 1 Introduction

X-linked hypophosphatemic (XLH) rickets is a rare, lifelong, progressive metabolic bone disease, affecting approximately 1 in 20,000–60,000 people worldwide (1, 2). It is caused by mutations in the phosphate-regulating endopeptidase homologue on the X chromosome (*PHEX*) gene that lead to an increase in fibroblast growth factor 23 (FGF23) levels, which causes renal phosphate wasting, reduced intestinal phosphate absorption and low active vitamin D, ultimately resulting in chronic hypophosphatemia (1, 3–5).

The onset of the disease is usually in childhood, manifesting with progressive musculoskeletal deficits, including rickets, lower limb deformities, impaired growth, muscle weakness, pain and fatigue (1, 4, 5). XLH presents differently throughout the lifespan. Adults commonly experience unresolved complications from childhood, along with new cumulative deficits such as osteoarthritis, enthesopathies and spinal stenosis, leading to pain, stiffness and a decrease in physical activity, with an associated reduction in quality of life (4, 5).

Patients with XLH may also suffer from hearing loss, tinnitus and vertigo (4). Moreover, dentine and periodontal abnormalities of both deciduous and permanent teeth may occur without evidence of trauma or dental decay, causing recurrent periapical abscesses with fistulae (6–8).

Introduced in the 1980s (PMID: 6252463), the conventional treatment for XLH has been daily oral phosphate salts and active vitamin D metabolites, e.g. calcitriol and alfacalcidol (3, 4, 9). This treatment regimen partially compensates for renal phosphate wasting and deficient vitamin D activation thereby improving phosphate supply for bone mineralization (9, 10). Although clinical benefits are seen, especially if supplementation is started early in infancy, conventional treatment does not address the underlying pathophysiology of XLH and may be associated with adverse effects, such as hyperparathyroidism and nephrocalcinosis in many patients, especially with long-term use (4, 11).

Burosumab, a fully human monoclonal antibody, targets the excess FGF23 activity of XLH, thereby increasing renal phosphate reabsorption and enhancing intestinal phosphate absorption through the production of 1,25-dihydroxyvitamin D, the active form of vitamin D (4, 12). Burosumab improves phosphate homeostasis in children with XLH by increasing serum phosphate levels, leading to improvement of the disease manifestations characteristic of XLH with amelioration of skeletal and muscular complications, and reduced disability (12–15).

Burosumab received conditional approval from the European Medicines Agency (EMA) in February 2018, for treatment of XLH in children 1 year of age and older with radiographic evidence of bone disease, and adolescents with growing skeletons (4). Approval was based on two Phase II studies, which evaluated 52 children with XLH aged 5–12 years (UX023-CL201; 13) and 13 children with XLH aged 1–4 years (UX023-CL205; 14). By September 2020, burosumab had gained full approval from the EMA and the indication was updated to include children and adolescents aged 1−17 years with radiographic evidence of bone disease and adults (16). The update was based on a Phase III trial in 61 children with XLH aged 1–12 years for 64 weeks (UX023-CL301; 15, 16) and Phase III studies in adults (16–18). While the clinical trials included children with XLH aged 1–12 years and adults from 18 years old, they did not include adolescents aged 13–17 years.

Despite the relatively recent introduction of burosumab for XLH, real-world data is accumulating for its safe and effective use in clinical practice. A study on 52 children aged 5–12 years (19) has shown that, compared to conventional treatment, burosumab administration for 160 weeks can improve phosphate homeostasis and reduce total Rickets Severity Score (RSS) by -0.9 ± 0.1 (p<0.0001). In a study of 61 children with XLH (20), aged 1–12 years, burosumab treatment for 64 weeks was associated with improvements in phosphate metabolism compared to conventional treatment, as well as with reductions in total RSS by -0.9 in younger children under 5 years old (n=26) and by -1.4 in children aged 5–12 years (n=35). Transition from conventional therapy to burosumab therapy in 12 children and adolescents aged 1–18 years with XLH (21) was associated with an increase in mean serum phosphate from 2.6 mg/dL to 3.4 mg/dL 4 weeks after starting burosumab (p=0.004), which was sustained at 12 months post-burosumab (mean serum phosphate 3.5 mg/dL; p<0.001). Average RSS derived from lower-extremity radiographs were reduced from 3.0 prior to transition from burosumab to 1.4 after 24 months following transition (p<0.001).

The available outcomes have prompted the development of clinical practice guidelines for the use of burosumab in children with XLH, from 1 year old to puberty (closure of growth plates confirmed on bone X-ray) (22). Guidance on the use of burosumab in older children and adolescents, and how to manage the transition to adulthood is still lacking. Despite recommendations for the use of burosumab within the Summary of Product Characteristics (SmPC) (16) and published guidelines (4), a survey of 20 European doctors (see *Methodology*) has indicated that initiation of treatment with burosumab, decisions to switch therapy, and dosing are handled differently based on individual clinician experience, with some national differences becoming apparent. This may reflect differences in selection criteria, patient demographics and approaches to treatment, from both individual and national perspectives, and has direct implications for patient selection, starting dose, dose titration and disease-monitoring practices.

Based on these differences and the limited guidance information for the use of burosumab in patients with XLH, a group of medical experts from various European centers sought to document the different practices and their experience associated with the management of children and adolescents with XLH treated with burosumab, including the transitioning of patients from adolescence to adulthood.

The aim of this article is to provide expert-led insights on the use of burosumab in children and adolescents with XLH in Europe, highlight the research gaps and the paucity of data to inform the ongoing management of adolescents and young adults with XLH, and provide suggestions and recommendations to address points raised.

## 2 Methodology

To objectively explore potential differences in prescribing behavior concerning the treatment of children with XLH with burosumab, a preliminary survey was conducted through a third party involving 10 doctors from Germany, 5 from France and 5 from the UK. To ensure participants had sufficient experience to meaningfully complete the survey, they had to be personally responsible for treating a minimum number of XLH patients with burosumab.

The survey comprised a 30-minute, moderated, web-assisted, in-depth interview. Anonymized dosing data collected through patient record forms documenting the last patients seen in clinic treated with burosumab (3–5 per participant) was used to support discussion. Each participant was given three hypothetical patient cases and asked how they would prescribe burosumab in terms of starting dose, titration and treatment goals. Hypothetical cases are provided in **Appendix 1**. The results from this preliminary survey revealed national and individual differences in prescribing behavior.

We (the authors of this paper) were then convened to form an expert practice exchange group to comment on the data generated by the survey, drawing on our experience with the use of burosumab in treating patients with XLH. We are Europe-based clinical experts specialized in pediatric and adult endocrinology, nephrology and metabolic bone diseases from seven countries (**Table 1**). Experts with experience managing adult patients with XLH were also included to facilitate discussion on transition of XLH children to adult services.

**Table 1:** Expert practice exchange group participants (all authors of this paper).

Author	Country	Center	First managed patients with XLH	First prescribed burosumab for patients with XLH	Patients with XLH managed	Patients with XLH managed with burosumab
			Year		Estimated total number (estimated number pediatrics)	
Adalbert Raimann	Austria	Vienna	2011	2019	30 (24)	8 (8)
Agnès Linglart	France	Paris	2001	2014	200 (180)	60 (60)
Dirk Schnabel	Germany	Berlin	1990	2016	50 (42)	25 (20)
Giampiero Baroncelli	Italy	Pisa	1982	2018	91 (60)	14 (14)
Andrea Magnolato	Italy	Trieste	2017	2019	10 (4)	-*
Carmen de Lucas-Collantes	Spain	Madrid	2009	2018	8 (8)	5 (5)
Fernando Santos	Spain	Oviedo	1991	2017	6 (6)	1 (1)
Ola Nilsson	Sweden	Örebro and Stockholm	2009	2018	20 (20)	7 (7)
Nick Shaw	UK	Birmingham	1990	2016	70 (70)	25 (25)
M. Zulf Mughal	UK	Manchester	1985	2018	75 (75)	20 (20)

*Before 2019, all conventional therapy; now, almost all burosumab.

XLH, X-linked hypophosphatemic rickets.

**Figure 1:**
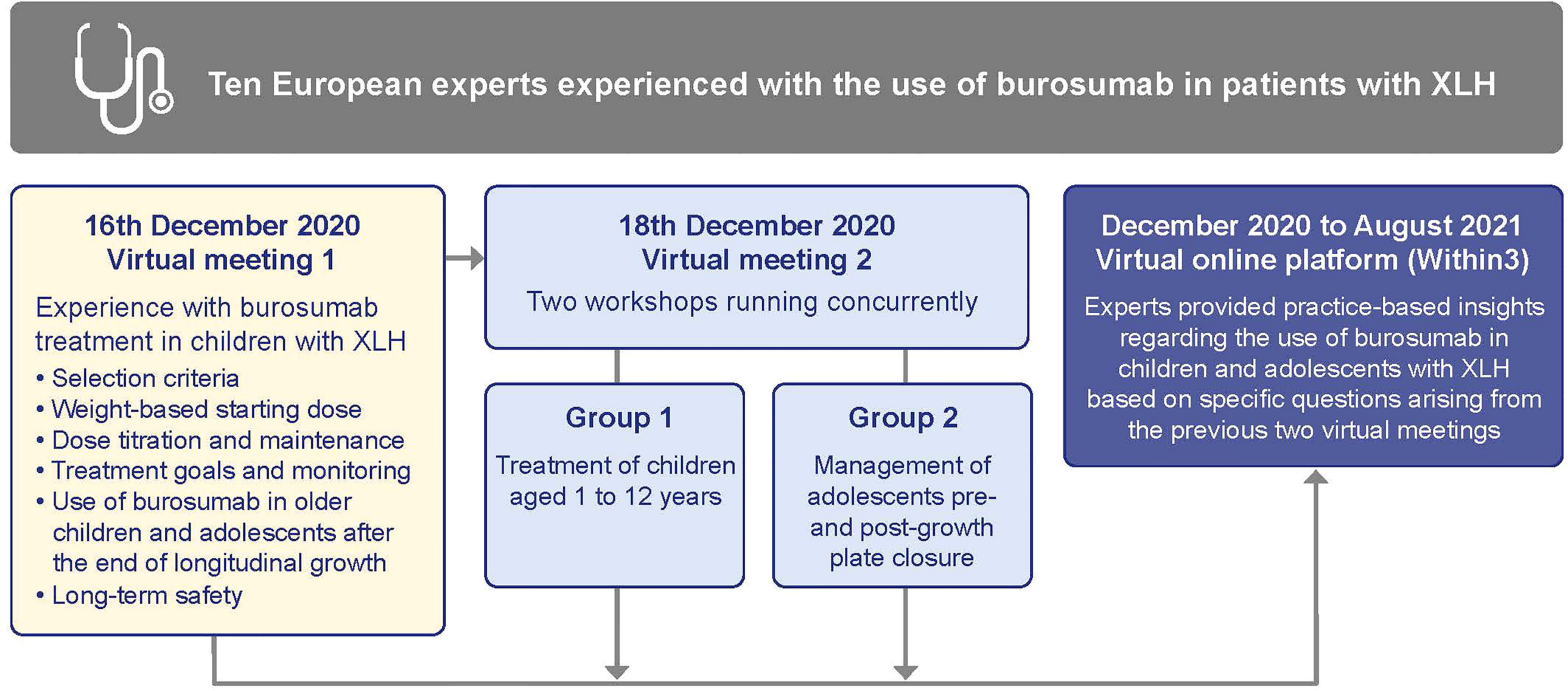
Format of the Expert Practice Exchange meetings. XLH, X-linked hypophosphatemia.

We met at two virtual working sessions in December 2020, firstly to discuss the data in the context of our own personal experience and, secondly to discuss what actions, if any, would be appropriate to provide consistency in treatment practice across Europe (**Figure 1**). While these meetings were organized and funded by the medical department of Kyowa Kirin, the company had no involvement or influence in any of our discussions or outputs.

We then used an online collaboration platform, Within3 (https://www.within3.com/), to collaboratively address key questions raised during the two working sessions, and provide suggestions on selection criteria, starting dose, dose titration and treatment monitoring in children and adolescents with XLH treated with burosumab. We iteratively contributed to suggestions by answering a series of questions (provided in **Appendix 2**) in full transparency with each other, and by replying to comments and questions from others in the group in real time.

This article represents the outcomes from the virtual and collaborative working sessions, and aims to summarize our experience and thoughts concerning the use of burosumab in children and adolescents with XLH at this early stage in the medicine’s availability in Europe.

## 3 Results

### 3.1 Use of burosumab in growing children

#### 3.1.1 Criteria for burosumab treatment

At the time of our meetings, criteria for selecting children with XLH for burosumab treatment varied between European countries and were often based either on eligibility criteria for early access programs (for example, in Spain from 2018 to 2020) or qualification for reimbursement from health insurance or governmental payer systems, rather than clinical opinion. In some countries, burosumab was reimbursed for all children with XLH over 1 year who were still growing, whereas in other countries, reimbursement was only for children with a more severe XLH phenotype who were still growing. In Austria, France and Sweden, criteria were dependent on disease severity or on inadequate response or complications to conventional treatment. In these countries, conventional treatment was the designated first-line treatment and only patients with overt rickets, despite adequate conventional therapy, or poor response and/or complications to conventional therapy, qualified for burosumab treatment. For example, in Sweden, a national XLH expert committee is evaluating the indication for burosumab treatment in each patient that are being considered to switch from conventional to burosumab therapy.

In contrast, in Germany, the UK and Italy, all children above 1 year with a confirmed diagnosis of XLH, radiographic evidence of bone disease and a growing skeleton were eligible for first-line treatment with burosumab (**Table 2**).

**Table 2:** Criteria for burosumab treatment for children and adolescents with XLH in Europe, as of September 2021.

Country	Criteria for burosumab treatment*
**Austria**	Patients with XLH treated with CT for a minimum of 6 months who have poor response^†^ and/or complications to CT^‡^ There are single case exceptions to this criterion
**France**	Patients with XLH treated with CT for a minimum of 12 months who have a poor response^†^ and/or complications to CT Patients with XLH and overt rickets, including naïve patients with a significant delay in diagnosis
**Germany**	Genetically confirmed XLH (*PHEX* mutation)
**Italy**	Children >1 year old with confirmed XLH (*PHEX* mutation or FGF23 >30 pg/mL) with radiographic evidence of bone disease (RSS >1.5) and growing skeleton until cessation of growth (AIFA guidance). Although burosumab is licensed for adults, it is not reimbursed; however, compassionate use for patients >18 years old is possible
**Spain**	All four of the following criteria specified in the pharmacoclinical protocol for the use of burosumab by Spain’s National Health System published in January 2021 must be met: 1) Children >1 year old and growing adolescents with a confirmed diagnosis of XLH (genetic confirmation is not specified). 2) Treatment with oral phosphate and active vitamin D for at least the previous 12 months, at appropriate doses, at specialized referral centers. 3) Radiographic evidence of bone disease and RSS ≥2 total score. 4) Epiphyseal closure has not occurred
**Sweden**	Patients with XLH treated with CT who have poor response^†^ or overt rickets despite appropriate dose of CT Each case evaluated by a national clinical committee
**UK**	Children with XLH >1 year old until cessation of growth, with radiographic evidence of bone disease (NICE guidelines)

*Inclusion criteria for Early Access Programme and/or reimbursement when commercially available. ^†^No globally accepted, validated criteria for assessing disease severity or treatment response exist; our discussions suggest centers approach this in their own way. ^‡^Criteria under ongoing re-evaluation.

AIFA, Agenzia Italiana del Farmaco (Italian Drug Regulatory Authority); CT, conventional treatment; FGF23, fibroblast growth factor 23; NICE, National Institute for Health and Care Excellence; *PHEX*, phosphate-regulating endopeptidase homolog on the X chromosome; RSS, Rickets Severity Score; XLH, X-linked hypophosphatemia.

The expert consensus from our survey is that burosumab treatment should be started as early as possible in children with XLH aged 1 year or older, particularly in those with RSS ≥2 (**Box 1**).

Box 1 **Experts’ suggestions for the use of burosumab in children and adolescents with XLH**

**Table:** 

1.	If guidelines and reimbursement criteria allow, burosumab treatment should be started as early as possible in children with XLH over the age of 1 year, particularly in those with profound rickets (RSS ≥2).* If conventional therapy is used as initial treatment, a prompt switch to burosumab is required in cases of insufficient response
2.	Serum phosphate levels below the age- and sex-specific LLN may be acceptable if there is a sustained decrease in ALP, improvement of rickets and the patient is responding clinically
3.	Children who have started treatment with burosumab should continue treatment throughout adolescence
4.	Changes in physical ability and quality of life in older children may require the use of monitoring tools similar to those used in the adult population with XLH

*An ongoing study is currently investigating early treatment with burosumab, the results of which will provide information on the optimal timing of when to start treatment with burosumab (23).

ALP, alkaline phosphatase; LLN, lower limit of normal; RSS, Rickets Severity Score; XLH, X-linked hypophosphatemia.

#### 3.1.2 Weight-based starting dose

Most of the expert group used the starting dose of 0.8 mg/kg of burosumab rounded to nearest 10 mg, administered subcutaneously every two weeks (Q2W), in accordance with the European SmPC, to a maximum dose of 90 mg (16). However, in Austria and the UK, a starting dose of 0.4 mg/kg Q2W was used by some experts, in line with the international consensus statement issued in 2019 or local country guidelines, such as the National Institute for Health and Care Excellence (NICE) and the British Paediatric and Adolescent Bone Group (BPABG) (4, 12, 24).

#### 3.1.3 Dose titration and maintenance

Given the phenotypical and physiological variations in XLH (e.g. in FGF23 levels, phosphate levels and degree of skeletal involvement), it is expected that, for individual patients, different doses of burosumab may be required to provide optimal clinical outcome, which is why dose titration is recommended (16). We all used fasting serum phosphate levels to guide titration, titrating doses to achieve levels above age- and sex-specific lower limit of normal (LLN) reference values defined by local laboratories. In many countries, it is standard practice to modify the burosumab dose only after: 1) at least two injections at the same dose had been given; and 2) fasting serum phosphate level remained below the LLN.

Titrated doses may be linked to age (related to growth spurt) and/or disease severity, based on our experience and the European SmPC. For example, adolescents and children with severe disease may require titration to higher doses to normalize serum phosphate and maintain clinical improvement. In recently published recommendations on the use of burosumab in children and adolescents, French experts proposed a target of serum phosphate levels of 1.2 mmol/L or above for all children <16 years old and 1.0 mmol/L for teenagers ≥16 years (25). In addition to fasting serum phosphate levels, physicians also use the ratio of tubular maximum reabsorption of phosphate to glomerular filtration rate (TmP/GFR) and evolving alkaline phosphatase (ALP) measurements to assist in titration dosing decisions.

We also discussed the current presentation of burosumab, which is available in single vials containing 10/20/30 mg burosumab in a 1 mL solution for injection, and whether more flexibility in vial size is required (16). We agreed that availability of a 5 mg vial may facilitate titration of burosumab and would be particularly useful in the dosing of young infants e.g. a 1-year-old with XLH.

#### 3.1.4 Treatment goals and monitoring

As there were no globally accepted, validated criteria for scoring the severity of XLH or assessing treatment response, part of this exercise was to understand how clinicians treating patients set treatment goals and evaluate response to treatment. Regardless of severity, our primary goals with burosumab treatment were the same, i.e. to decrease bone pain, heal rickets and improve growth. As the serum phosphate requirements change during childhood and adolescence, it is important to consider the age- and sex-specific reference values during dose titration (4, 26).

We agreed that serum ALP, as well as serum phosphate, has an important role in monitoring the progress of children with mild and severe forms of XLH. We proposed performing serial measurements of ALP levels to establish the trend of ALP over time. We also suggested that a consistent rise in serum ALP above the age- and sex-specific upper limit of normal of the reference range may warrant an increase in the burosumab dose, whereas a continued decline in serum ALP, despite still being above the age- and sex-specific upper limit of normal, may indicate an adequate physiological and clinical response. In this context, the expert group recommends that serum phosphate levels below the age- and sex-specific LLN may be acceptable as a treatment response, if there is a sustained decrease in ALP, improvement of rickets and the patient is responding clinically (**Box 1**).

We considered clinical improvement in the skeletal disease, irrespective of normalization of biochemical markers, to be an additional indicator of treatment response. We suggested the incomplete healing of rickets or lack of improvement upon periodic imaging (radiographs or magnetic resonance imaging [MRI] scans) of the knee/standing long leg may indicate the need to increase the burosumab dose. At the time of writing, in Italy, for patients receiving burosumab, it is mandatory to assess RSS after 12 months of treatment to demonstrate its efficacy on rickets lesions and continue treatment with reimbursement. We agreed that changes in bone deformity may also represent an important parameter to assess the outcomes of burosumab treatment, but that, in severe cases of XLH, an improvement in rickets assessed by imaging may not necessarily be associated with an improvement in leg bowing. Consequently, the expert recommendation is that changes in physical ability and quality of life in older children being treated with burosumab should be monitored using tools similar to those used in the adult population with XLH (**Box 1**).

### 3.2 Use of burosumab in adolescents and young adults after growth cessation

As for younger children, members of our group used biochemical analyses of serum phosphate, 25-hydroxyvitamin D (25(OH)D), calcium, parathyroid hormone (PTH) and TmP/GFR to monitor the treatment response to burosumab in adolescents and young adults with XLH. Identifying and treating vitamin D deficiencies using periodic measurements and supplementation was deemed important due to the widespread nature of such deficiencies in Europe and a decline in vitamin D levels in some XLH patients during burosumab treatment (16, 27). It should be noted that each country has its own recommendations on vitamin D intake to prevent vitamin D deficiency; and clinicians should refer to their own country’s recommendations. We recommend maintaining serum 25-hydroxyvitamin D >50 nmol/L (20 ng/mL) to prevent secondary hyperparathyroidism and associated phosphaturia. Adequate body stores of vitamin D may also facilitate burosumab mediated 1,25-dihydroxyvitamin D synthesis.

We established that adolescents with XLH treated with burosumab may require different tools to monitor treatment response and clinical outcomes than those used for younger children. Notably, the healing of rickets monitored by radiographs becomes less sensitive as children approach growth cessation and epiphyseal fusion (28). Decline in growth velocity, bone age and radiological closure of growth plates at the wrist and knee are sufficient to monitor growth cessation and were the methods used most frequently by members of our group in clinical practice. MRI could also be a clinically relevant tool for determining closure of growth plates in adolescents (29) and assessing improvements in bone quality after treatment by looking at rachitic abnormalities, and for the presence of Harris lines and bone marrow abnormalities of the epiphysis (30, 31), although it was not used by all clinicians in our group for this purpose.

In terms of patient-reported outcomes, we agreed that changes in physical ability and quality of life in adolescents could be monitored with tools similar to those used in the adult population with XLH, e.g. 6-minute walk test, handgrip strength, Brief Pain and Fatigue Inventories and the Western Ontario and McMaster Universities Arthritis Index (WOMAC).

Our discussions revealed that, in many European countries, burosumab treatment was discontinued once longitudinal growth ceases, which can be as early as 12–14 years of age, because of a gap between regulatory approval and reimbursement of medicines. We discussed whether it was more appropriate to use chronological age or physiological age−based parameters, such as growth plate closure, to determine when to change the burosumab dosing regimen from the pediatric Q2W to adult dose every four weeks (Q4W) and agreed that physiological age is the more appropriate indicator.

Our talks highlighted that there have been no clinical studies of burosumab specifically in adolescents aged 13–17 years, leaving a critical gap in how to deal adequately with this patient population. Given the data on burosumab safety in adolescents up to 18 years, our expert view is that children who have been started on burosumab should continue treatment throughout adolescence (**Box 1**).

### 3.3 Safety of burosumab treatment

We all agreed that burosumab is well tolerated, with few and mostly mild side effects that rarely caused cessation of treatment. The most commonly observed side effect was mild injection site reaction, which resolved without affecting long-term treatment.

## 4 Discussion

Given the rarity of XLH and the relatively recent introduction of burosumab as a treatment for this genetic condition, there is a lack of peer-reviewed published information that can be used by clinicians as a guide to treatment. To address this, we convened, as an expert practice exchange group with clinical experience with burosumab, with the aim of exchanging experiences and publishing these practice-based insights on the use of burosumab in children and adolescents with XLH. The key points of discussion related to patient selection criteria, starting dose of burosumab, dose titration and treatment monitoring.

The variable selection criteria reported between countries, is reflective of experience with many other new medicines for rare disorders, where decisions about access depend not only on rigorous evaluation of clinical evidence by regulatory bodies such as the EMA, but also on robust, national health technology assessments of broader social, economic, organizational and ethical factors (32, 33).

It is evident that the differences in selection criteria of children with XLH may have implications on the starting dose of burosumab, dose titration and maintenance dosing as they depend on the patient’s age and disease severity (16). When burosumab was first approved in Europe in 2018, the starting dose was 0.4 mg/kg given subcutaneously Q2W (4). In countries such as the UK, where all children above 1 year are eligible for burosumab treatment, we proposed 0.4 mg/kg of body weight as a starting dose may be adequate for children with milder forms of XLH and could be sufficient as a maintenance dose in select patients who continue to show improvement in clinical outcomes (12). However, in countries where selection criteria excluded children with milder forms of XLH, we proposed a starting dose of 0.4 mg/kg would likely be insufficient to obtain fasting serum phosphate levels above the LLN. In August 2019, the European SmPC for burosumab was updated and the recommended starting dose changed from 0.4 mg/kg to 0.8 mg/kg of body weight given subcutaneously Q2W (16).

Guidance on the titration of burosumab to achieve levels within the reference range for age, with a target of LLN to decrease the risk for ectopic mineralization, is given in the SmPC (16): If fasting serum phosphate is below the reference range for age, the dose may be increased stepwise by 0.4 mg/kg up to a maximum dose of 2.0 mg/kg or up to a total dose of 90 mg, with periods between adjustments being at least 4 weeks (16).

The biomarkers most commonly used by members of our group to guide burosumab titration were serum fasting levels of phosphate and ALP, and measures of renal phosphate handling such as TmP/GFR. Interpretation of laboratory values to guide dosing depends on the availability of evidence-based reference values that are appropriate for the patient’s physiological status. Several physiological factors, including age, sex and pubertal development, can cause changes in analyte values (34). This is especially important in children and adolescents, where growth and development may significantly impact the phosphate requirements and, therefore, the recommended reference ranges (35). In addition, reference ranges for the same biomarker may vary based on the different laboratories and analytical platforms being used (36). In the burosumab treatment of patients with XLH, the lack of harmonization of age- and sex-specific reference ranges for different biochemical markers makes it difficult to recommend a standard target value or measurement to aim for. We suggested clinicians consult their local laboratories for advice, and recommend the development and dissemination of robust local reference ranges.

While we agreed the overall goal of XLH treatment is to heal rickets in children and improve osteomalacia in older children who have stopped growing and in adults, the initial indicator of treatment response with burosumab, based on its mechanism of action, is to increase serum phosphate levels, regardless of severity, age and sex. The SmPC advises targeting phosphate levels to the LLN (16).

In clinical trials of both children and adults with XLH, burosumab consistently increased serum phosphate levels (13–15, 17, 18). In the pediatric clinical trials, the burosumab doses were titrated to achieve serum phosphate trough levels above LLN. Data from these trials suggest that titrating burosumab to achieve serum phosphate levels at around LLN resulted in the normalization of TmP/GFR, a reduction in the bone metabolic marker serum ALP, and healing of rickets, with improvement in bone mineralization (13–15, 17, 18). Although these clinical trials used a serum phosphate threshold lower than the age- and sex-specific LLN, particularly in younger children, patients responded clinically. In clinical practice, there have been some reports of children and adolescents with XLH who do not achieve serum phosphate levels above the age- and sex-specific LLN with variable outcomes (36, 37). However, in a sub-group analysis of the Phase III trial of burosumab in adults with XLH, clinical outcomes improved in patients treated with burosumab compared with placebo, including those who achieved the LLN of serum phosphate at fewer than half of their study visits (17, 38). We agreed that sustained improvement of serum phosphate levels could be a good indicator of clinical effect of burosumab in children with XLH, even if individual measurements do not attain the age- and sex-specific LLN, and a decline in serum phosphate levels may be an alert to review the dose of burosumab.

Other measurements used by members of our group alongside serum phosphate to indicate responses to burosumab include TmP/GFR and serum ALP. We suggested single measurements of serum phosphate should not be used as the only parameter to indicate treatment response or clinical outcomes in patients, especially in patients that fail to reach the normal reference range. Instead, we proposed serial measurements should be used to demonstrate persistent improvement in serum phosphate levels as a change from baseline, together with changes in TmP/GFR, ALP and other signs of clinical response, e.g. improvements in bone pain, rickets and growth.

In children or adolescents with XLH who do not achieve the targeted age- and sex-specific LLN of serum phosphate, despite titration to the highest dose of burosumab (2 mg/kg or a total dose of 90 mg), we suggest investigating other underlying causes of hypophosphatemia on a case-by-case basis. It is important to review the details, including evidence of malnutrition, hungry bone syndrome and hyperparathyroidism. Increased PTH can cause further urinary phosphate wasting and, if serum PTH is raised, it is important to ensure serum 25(OH)D is maintained above 50 nmol/L and the child receives adequate amounts of dietary calcium (4, 39). Another area to consider is whether bioavailability of burosumab could be compromised by factors that may affect the subcutaneous absorption of the drug, e.g. injection technique and lipohypertrophy (40).

We established that dosing changes need to be managed as children grow and monitoring of body weight is required to ensure there is no inadvertent decrease in the burosumab mg/kg dose as the child gains weight. We suggested serum phosphate concentrations below the age- and sex-specific LLN may be acceptable and the same maintenance dose could be considered if: 1) the serum ALP is normal; 2) there is evidence of rickets healing; 3) attainment in age-related growth velocity; and 4) the patient is responding well clinically.

Members of the expert group assessed clinical improvement in skeletal disease in children with XLH by the healing of rickets using periodic radiographic imaging. Although the RSS is an important parameter used to verify XLH severity, we agreed it may be less useful in patients affected by milder forms of rickets and is rarely used outside the clinical trial setting. Moreover, the monitoring of rickets healing using RSS and radiographs becomes less sensitive in children >13 years old or once growth plate closure has occurred (28, 41, 42). MRI could be helpful to determine closure of growth plates in adolescents and to monitor changes in bone quality (43, 44). Additional tools such as EOS 2D/3D imaging and Gait lab could be useful to determine changes in bone quality and bone deformity, especially in older children (4, 45).

We believe that, in adolescents and young adults after growth plate closure, there is a need to establish suitable biochemical markers due to changes in bone physiology. Serum ALP may be less sensitive to evaluate healing of osteomalacia in adolescents and young adults following growth plate closure, because most adults with XLH, with clinical symptoms of osteomalacia, have normal levels of serum ALP (46). In these patients, bone-specific ALP or other markers of bone turnover (e.g. osteocalcin, procollagen 1 intact N-terminal propeptide and C-terminal telopeptide of type 1 collagen) may be used as a complement to ALP (17, 18).

The group agreed that symptoms such as bone and muscular pain, fatigue and difficulty in walking, may be indicative of worsening osteomalacia and could be helpful as a guide to monitor treatment in adolescents. We recommended developing a composite osteomalacia severity score, which could be useful in late adolescence and during the transition phase, to assess progress and improvement with treatment. The development of such a score would require careful consideration as to the parameters to be measured (such as function, pain and quality of life), rationale of the best tools to measure these parameters and how to validate such a tool in a rare genetic condition.

Based on country-specific guidance shared during our meetings, it universally appears that burosumab treatment is stopped at growth cessation. This conflicts with our treatment goals as once treatment is stopped, osteomalacia may progress with worsening of disease. The skeleton is maturing and acquiring bone mass through this pivotal stage in development (47). We agreed it is likely that maintaining adequate serum phosphate during this period would be beneficial for long-term bone health and may have the potential to halt disease progression during this important period of bone acquisition. Controlled, long-term studies with continued burosumab treatment would be useful to assess its efficacy to prevent progression of bone disease during late adolescence and early adulthood. Before clinical studies, we suggested that, if accepted through national funding systems, it made clinical sense to allow patients, as deemed necessary, to continue burosumab treatment without cessation, past adolescence and into early adulthood. However, some of us reiterated the need for clinical studies to provide evidence for the continuation of burosumab in adolescents. These are summarized in **Box 2**.

Box 2 **Recommendations for future developments and research**

**Table:** 

1.	Developing a combined score of expected events, based on clinical, biochemical and radiological examination could be a useful tool to assess response to treatment with burosumab
2.	Developing a tool to quantify the severity of osteomalacia development could be useful in late adolescents and during the transition into adulthood
3.	A clinical trial in adolescents would be useful to determine how to best proceed with the change in both dosing and frequency of burosumab treatment before and after the end of skeletal growth and into young adulthood

According to the European SmPC, for patients continuing burosumab treatment as they transition from adolescence to adulthood, the dose should be changed to the adult dosing regimen at 18 years of age, with a starting dose of 1.0 mg/kg of body weight given Q4W, rounded to the nearest 10 mg (16). However, as the maximum dose in children is 2.0 mg/kg Q2W, this may translate to both a reduction in burosumab dose and frequency of injections. Alternatively, there is the potential for ‘over treatment’ of adolescents with closed growth plates who continue the pediatric Q2W dosing regimen until the age of 18 years.

Chronological age is used to define the transition between childhood and adulthood but, in the context of XLH, growth cessation and the achievement of final adult height may be a more appropriate indicator to change the burosumab dose from pediatric to adult dosing regimen, given the changes in requirements for phosphate based on skeletal growth and skeletal maturation during this time (48). However, bone mass continues to be accrued at a relatively high rate in the years immediately after final height has been achieved (28).

Based on discussions and clinical experience with burosumab, we made several clinical suggestions for the use of burosumab in children and adolescents with XLH as shown in **Box 1** and recommendations for further research as shown in **Box 2**.

## 5 Limitations

The views reflected in this paper are based on our experience, as a select group of European experts, on the use of burosumab in children with XLH, in addition to relevant data obtained from treatment guidelines. The survey methodology used to formulate the expert opinions reported used a structured approach, with predefined clinical scenarios and iterative rounds of responses. We acknowledge that other expert survey methodologies, such as the Delphi process (49), can be used to develop expert consensus on clinical needs, such that our chosen method may have introduced bias. Therefore, the opinions reported herein may not necessarily reflect those of experts in other regional centers or countries.

There is a variation in the indication for use, as well as regulatory and reimbursement constraints, for burosumab treatment in different European countries, which impacts treatment in all age groups.

Due to the EMA approval for burosumab use in adults with XLH in September 2020, and the lack of clinical data in children aged 13–17 years, we had limited experience in the management of XLH with burosumab for the transition of existing patients from adolescence to adulthood. New data are required to address this limitation.

Clinical practice recommendations for the diagnosis and management of XLH were published in 2019 to support clinicians (4). At the time of drafting the recommendations, only the initial pediatric Phase II study in children with XLH aged 5–12 years old (UX023-CL201) was considered with respect to burosumab treatment (4, 13). The published Phase III clinical data in children and adults (15–17), and the more recent regulatory approval for the use of burosumab in the treatment of adults and older adolescents with XLH were not available at the time of preparation of the recommendations (4). Therefore, real-world data from the XLH and post-authorization safety studies registries will be helpful to address modifications when updating the existing recommendations.

## 6 Conclusion

This expert group suggested that, if feasible, children with XLH should start treatment with burosumab as early as possible, from the age of 1 year, particularly if they have profound rickets (RSS ≥2), and should continue treatment without cessation throughout adolescence and where possible into adulthood, with the aim of improving clinical outcomes and slowing/halting the progression of disease.

We suggested that burosumab treatment should aim to provide a sustained increase in serum phosphate levels, and that the evaluation of serum phosphate and adjustment of burosumab treatment should be done in the context of other biomarkers and clinical assessments.

Following growth plate closure in adolescents with XLH, we agreed radiographic assessment of rickets is no longer relevant for monitoring improvements. Other measurement tools that are used to assess clinical and patient-reported outcomes in adults may also be reliable to monitor treatment in adolescents.

We believed that the current gap in knowledge on the use of burosumab and the optimal management of adolescents with XLH and the transition to adulthood warrants further investigation with a clinical trial.

We hope that the expert-led insights, suggestions and recommendations summarized in this paper may be considered as a useful addition to other published guidelines for the management of XLH until further real-world evidence becomes available to better understand the treatment of XLH in children and adolescents with burosumab and the transition to adult management.

## Author contributions

All authors contributed to manuscript revision, read and approved the submitted version.
