# Supplementary material for: Burosumab for X-linked hypophosphatemia in children and adolescents: Opinion based on early experience in seven European countries
Source: Front Endocrinol (Lausanne). 2023 Jan 31;13:1034580. doi: 10.3389/fendo.2022.1034580 (PMC9928183; doi:10.3389/fendo.2022.1034580)
Supplement: Supplementary file 1 [file DataSheet_1.docx]

**APPENDIX 1**

**HYPOTHETICAL PATIENT CASES USED DURING THE INITIAL 30-MINUTE SURVEY**

**CASE 1: TODDLER**

Sally is a 22-month-old female who was diagnosed with XLH at birth, as she was born to father with the condition. She has been treated with oral phosphate and activated vitamin D supplements since the age of 9 months. Sally has an older brother with XLH.

Auxology:

- Height: 76.5 cm; SDS -2.35 (0.4th centile)
- Weight: 10.46 kg; SDS -1.13 (9th to 25th)

Biochemical evaluation:

- Corrected calcium: 2.27 mmol/L (2.15–2.65)
- Inorganic phosphate: 0.78 mmol/L (1.1–2.0)
- Alkaline phosphatase: 840 IU/L (100–733)
- Parathyroid hormone: 6.6 pmol/L (1.1–6.9)
- 25-hydroxyvitamin D: 65 nmol/L (levels > 50 considered to be adequate)

Radiograph of left knee:

-
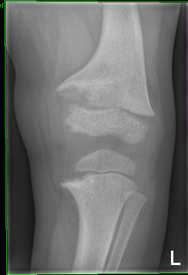
Parents are concerned about Sally’s delayed walking (20 months), small size, bowed legs and general irritability despite therapy.
- Radiograph of the left knee shows widening and fraying of metaphyses, consistent with inadequately treated rickets.Parents report that Sally hates the taste of the phosphate supplements and often complains of belly ache. They wish her to be switched from current treatment to burosumab.

**CASE 2: CHILD**

Ahmed is a 7-year-old boy who arrived in the UK with his family as a refugee from Aleppo, Syria. His parents are second cousins but there is no family history of rickets/osteomalacia. Ahmed was noted to have ‘bendy legs’ at the age of 2½ years. He has had recurrent dental abscesses which had been treated with oral antibiotics. He also complained of pain in his knees and ankles brought on by walking and physical activities.

At the age of 3¾ years, he was diagnosed as a case of nutritional rickets and treated with oral vitamin D3 (cholecalciferol) and calcium supplements. There was poor response to this treatment. The diagnosis of hypophosphatemic rickets was made when he was about 5 years old. Ahmed was treated with intermittent does of calcitriol and oral phosphate supplements while he was in a refugee camp, in Turkey.

Auxology:

- Age: 7 years and 2 months
- Height: 107.8 cm; SDS -2.48 (<0.4th centile)
- Weight: 24.0 kg; SDS 0.19 (25th -50th centile)

Biochemical evaluation:

- Corrected calcium: 2.41 mmol/L (2.20–2.70)
- Inorganic phosphate: 0.81 mmol/L (1.0–1.8)
- Alkaline phosphatase: 891 IU/L (60–320)
- Parathyroid hormone: 6.8 pmol/L (1.1–6.9 )
- 25-hydroxyvitamin D: 78.7 nmol/L (levels > 50 considered to be adequate)
- TmP/GFR 0.79 mmol/L: (1.10–1.88)
- Intact FGF23: 203 RU/ml (0–99)

Standing long-leg radiographs with close-up view of knees:


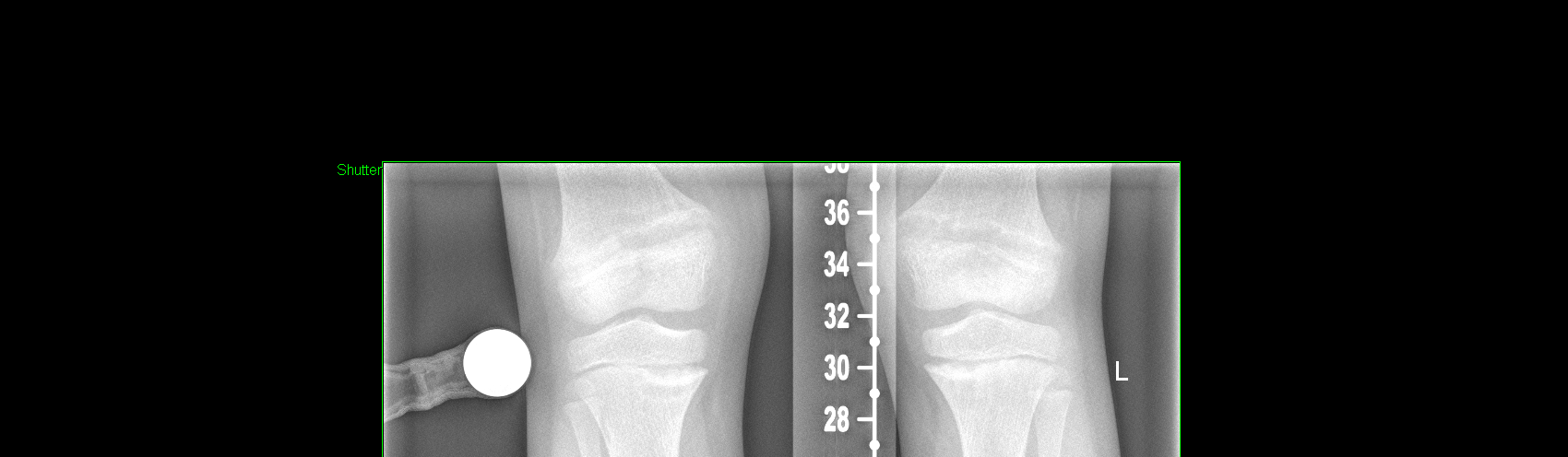

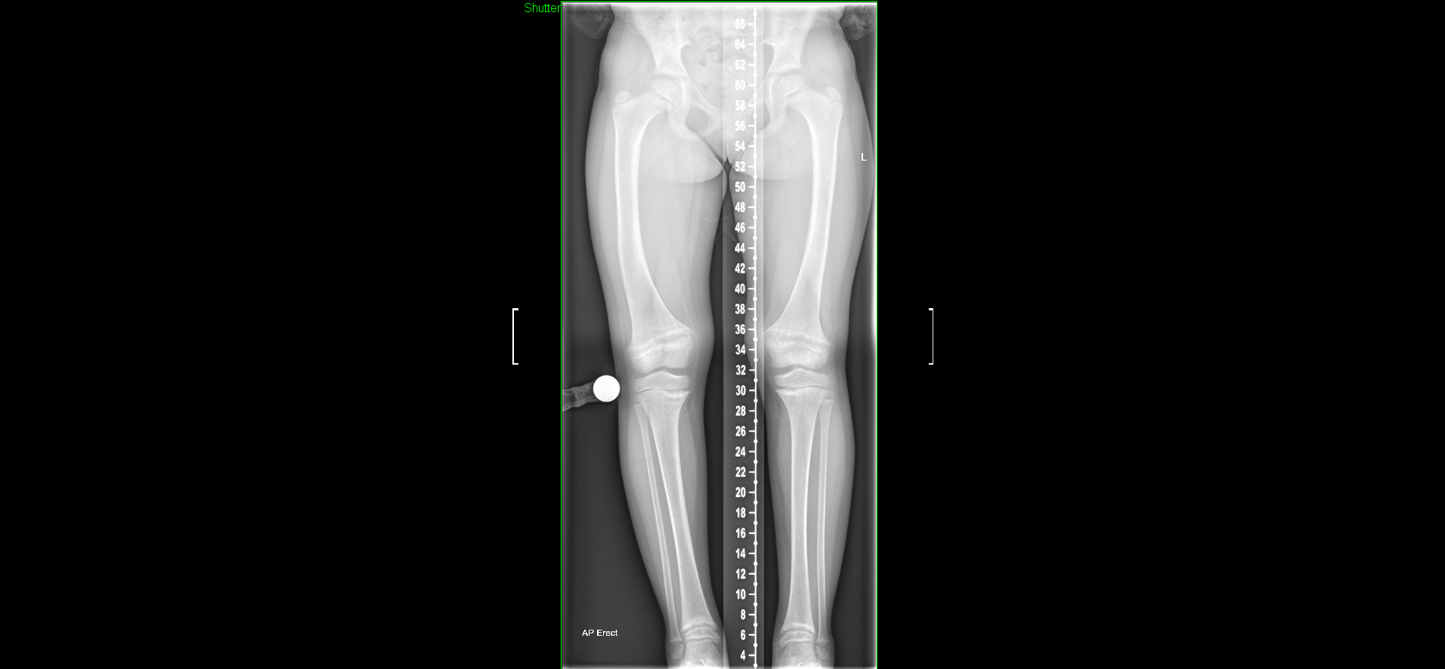


Genetic studies:

- Found to have a pathogenic mutation in the *PHEX* gene.
- Ahmed had mild scaphocephaly and his dentition was poor.
- He had a moderate degree of genu varum. His lower limb radiographs showed active rickets, with thickening of cortices with a coarse trabecular pattern in the metaphyses, which are characteristic radiological features of XLH.

Treatment:

- After discussion with the parents, you wish to start this patient on burosumab.

**CASE 3: TEENAGER**

John is 13-year-old male who was born to a mother with XLH. The diagnosis of XLH in John was made when he was about 3 years old. He was treated with ‘conventional treatment’ consisting of phosphate and activated vitamin D supplements. His adherence was sub-optimal. He developed severe genu varum. Therefore, he had epiphysiodesis to his knees and ankles in order to try and correct his lower limb deformities. His radiographs showed incomplete healing of rickets.

Assessment at the age of 9 years – on ‘conventional treatment’ since the age of 3 years

Auxology:

- Age: 9 years 8 months
- Weight: 22.75 kg; SDS -2.0 (0.4th & 2nd centile)
- Height: 111.5 cm; SDS -3.85 (which lies way below the 0.4th centile)

Biochemical evaluation:

- Corrected calcium: 2.24 mmol/L (2.15–2.65)
- Inorganic phosphate: 0.9 (1.0–1.8)
- Alkaline phosphatase: 469 IU/L (60–400)
- Parathyroid hormone: 40 pg/ml (15–65)


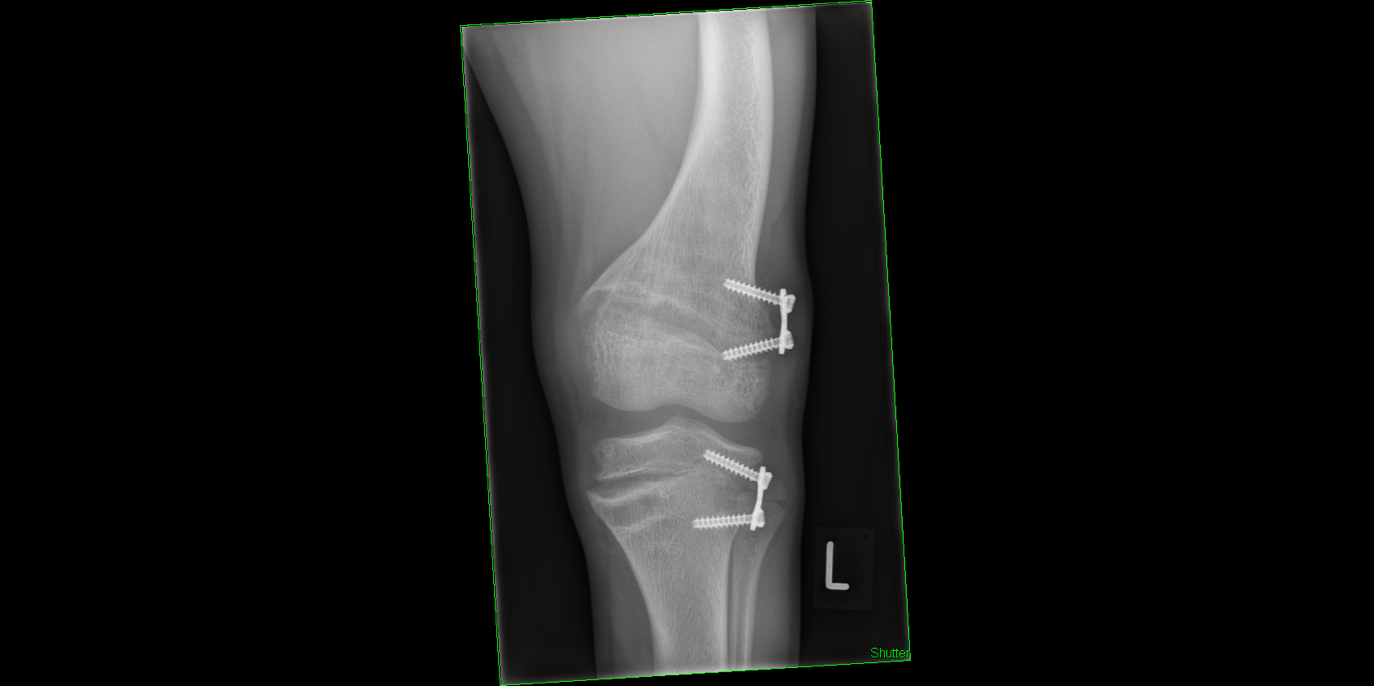
Radiographs:


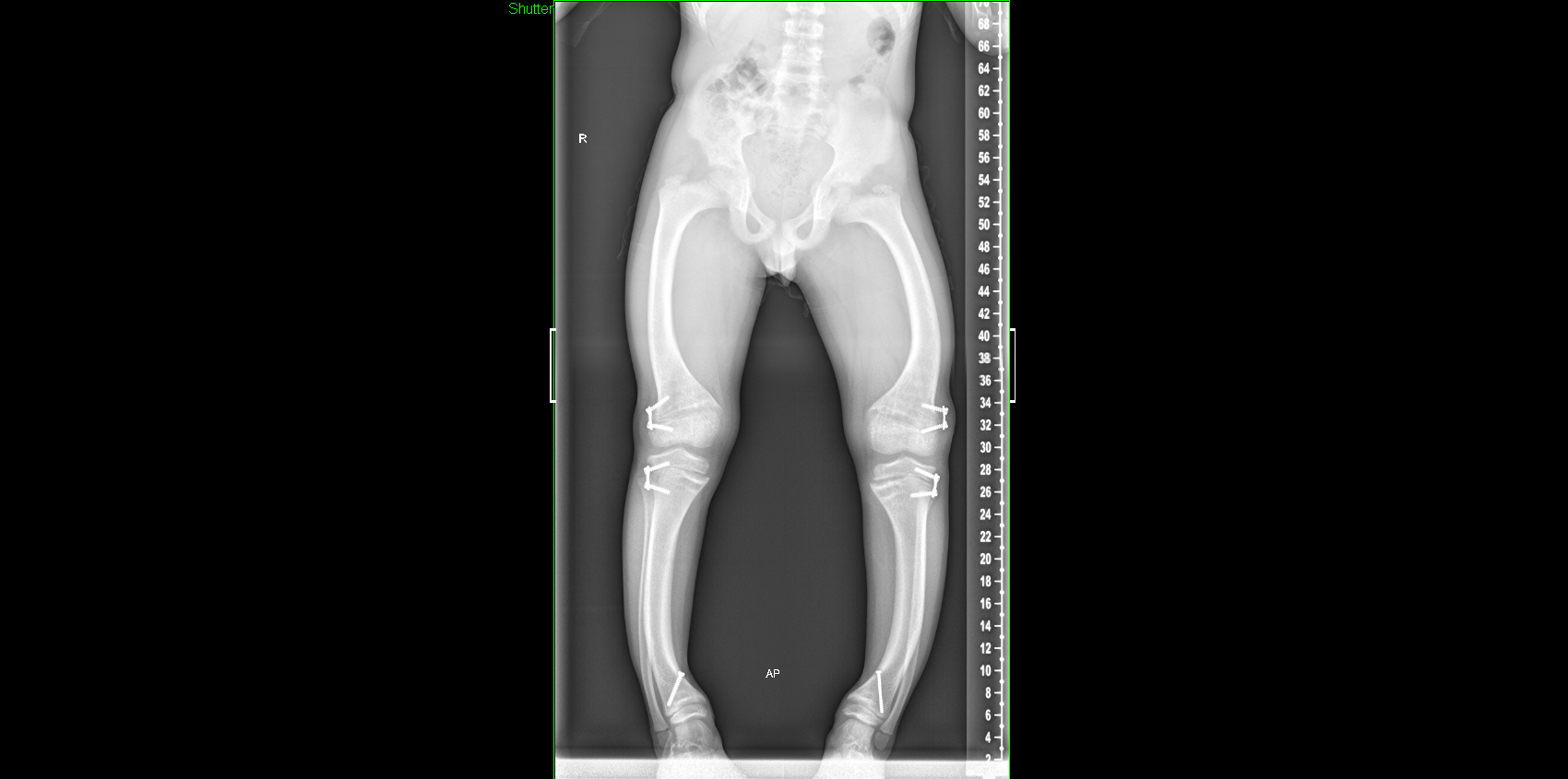
John was recruited into the Phase II trial of treatment of children with XLH with burosumab, just before his 10th birthday. The dose of burosumab was adjusted as mandated by the trial. At the end of the 64-week trial, treatment was continued and the dose of burosumab was adjusted to:

- Maintain his serum inorganic phosphate and TmP/GFR values within the lower end of the age-based reference range.
- Maintain his serum alkaline phosphatase within the reference range for his age.
- Radiographic healing of rickets.

**Assessment at the age of 13 years 8 months**

Auxology:

- Height: 130.6 cm; SDS -3.39 (which lies way below the 0.4th centile)
- Weight: 35.3 kg; SDS -1.65 (2nd centile)

Biochemical evaluation:

- Corrected calcium: 2.4 mmol/L (2.2–2.7)
- Inorganic phosphate: 0.99 (0.95–1.5)
- Alkaline phosphatase: 279 IU/L (60–400)
- Parathyroid hormone: 3.8 pmol/L (1.6–1.9)
- 25-hydroxyvitamin D: 50.7 nmol/L (levels > 50 considered to be adequate)
- TmP/GFR: 0.96 mmol/L (0.93–1.71)

Current medication:

- Burosumab 40 mg (1.2 mg/kg) injected subcutaneously, every fortnightly.
- What would be your treatment plan for this patient over the next 2–3 years?

Radiographs:


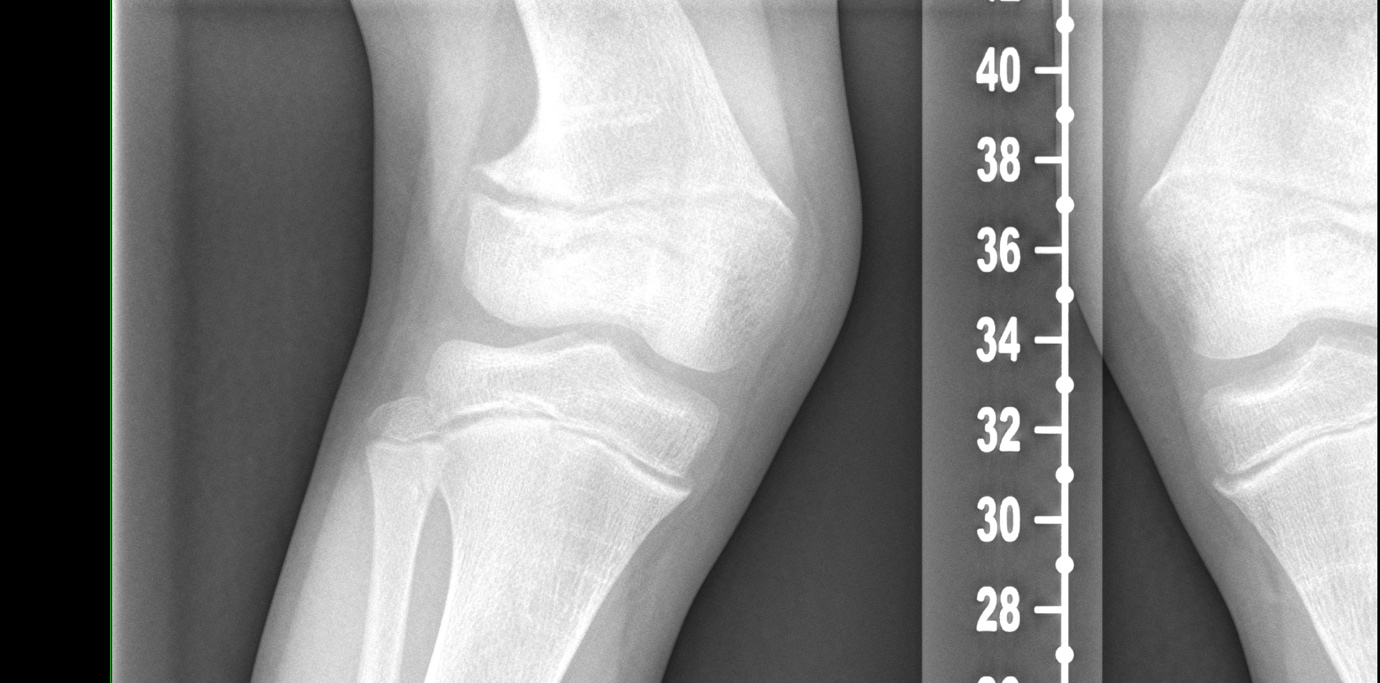
Radiograph shows healing of rickets but John’s genu varum persists.

**APPENDIX 2**

**Experience with burosumab in the management of children and adolescents with XLH**

From the previous meeting, the main areas highlighted in the treatment with burosumab in children and adolescents with XLH were:

- Dose titration and dose maintenance
- Treatment goals and monitoring, with a need to consider both biochemical and clinical differences in current practice
- The gap in the knowledge regarding the management of adolescents following growth plate closure and the transition of adolescents to the adult dosing regimen, including when and how this should be implemented.

Five questions have been prepared below that will help to develop a manuscript that will cover the early experience with burosumab in the management of children and adolescents with XLH in Europe and the UK

In your responses, please address how you would manage patients differently depending on the ages relevant to the discussion, i.e. toddlers and young children, older children nearing end-of-growth and adolescents post growth plate closure. The executive summary of the meeting in December 2020 as well as a manuscript outline are available on this platform for your reference.

1. There is variation in how people with XLH respond to treatment in general; therefore, a difference in dosing required is not unexpected. Based on the mechanism of action of burosumab, the initial goal of treatment is to increase serum phosphate and the SmPC advises targeting phosphate levels to the lower limit of normal (LLN). The dose can be increased from the starting dose to a maximum of 2mg/kg to achieve this target.
   1. In your opinion, what levels of serum phosphate should be targeted and why?
   2. What factors or characteristics affect the titration goal?
   3. What would you consider for patients that do not achieve the targeted serum phosphate levels?
2. Younger children compared with adolescents may require different tools to monitor biochemical and clinical outcomes:
   Radiological guides and rickets severity scores for monitoring may be less sensitive in adolescents and young adults with XLH
   Older children may require monitoring using alternative tools, similar to those used in the adult clinical trials of patients with XLH, to assess clinical outcomes and improvements in osteomalacia, physical ability and quality of life.
   1. Can you suggest which biochemical, clinical and imaging parameters/markers are most relevant to monitor progress in children of different ages with XLH on burosumab treatment?
3. Chronological age is used inconsistently to define end-of-growth but is seldom used in isolation to determine skeletal maturity and continued bone modelling may occur after final height has been achieved and the growth plates have closed. Lifelong bone health is also dependent on maximising peak bone mass (PBM) during the critical periods of growth, bone mineralisation and maturation. Childhood and young adulthood are particularly important for building PBM and is reported to be achieved in early adulthood.
   1. In your opinion, what is the best way to address the dosing regimen in the older child/adolescent nearing the end-of-growth?
